# Phosphatidylinositol 4-Phosphate 5-Kinases in the Regulation of T Cell Activation

**DOI:** 10.3389/fimmu.2016.00186

**Published:** 2016-05-13

**Authors:** Nicla Porciello, Martina Kunkl, Antonella Viola, Loretta Tuosto

**Affiliations:** ^1^Department of Biology and Biotechnology Charles Darwin, Istituto Pasteur-Fondazione Cenci Bolognetti, Sapienza University, Rome, Italy; ^2^Department of Biomedical Sciences, University of Padua, Padua, Italy; ^3^Venetian Institute of Molecular Medicine (VIMM), Padua, Italy

**Keywords:** PIP5K, actin cytoskeleton, T cell signaling, metabolism, CD28 co-stimulation

## Abstract

Phosphatidylinositol 4,5-biphosphate kinases (PIP5Ks) are critical regulators of T cell activation being the main enzymes involved in the synthesis of phosphatidylinositol 4,5-biphosphate (PIP2). PIP2 is indeed a pivotal regulator of the actin cytoskeleton, thus controlling T cell polarization and migration, stable adhesion to antigen-presenting cells, spatial organization of the immunological synapse, and co-stimulation. Moreover, PIP2 also serves as a precursor for the second messengers inositol triphosphate, diacylglycerol, and phosphatidylinositol 3,4,5-triphosphate, which are essential for the activation of signaling pathways regulating cytokine production, cell cycle progression, survival, metabolism, and differentiation. Here, we discuss the impact of PIP5Ks on several T lymphocyte functions with a specific focus on the role of CD28 co-stimulation in PIP5K compartimentalization and activation.

## Introduction

**Figure 1:**
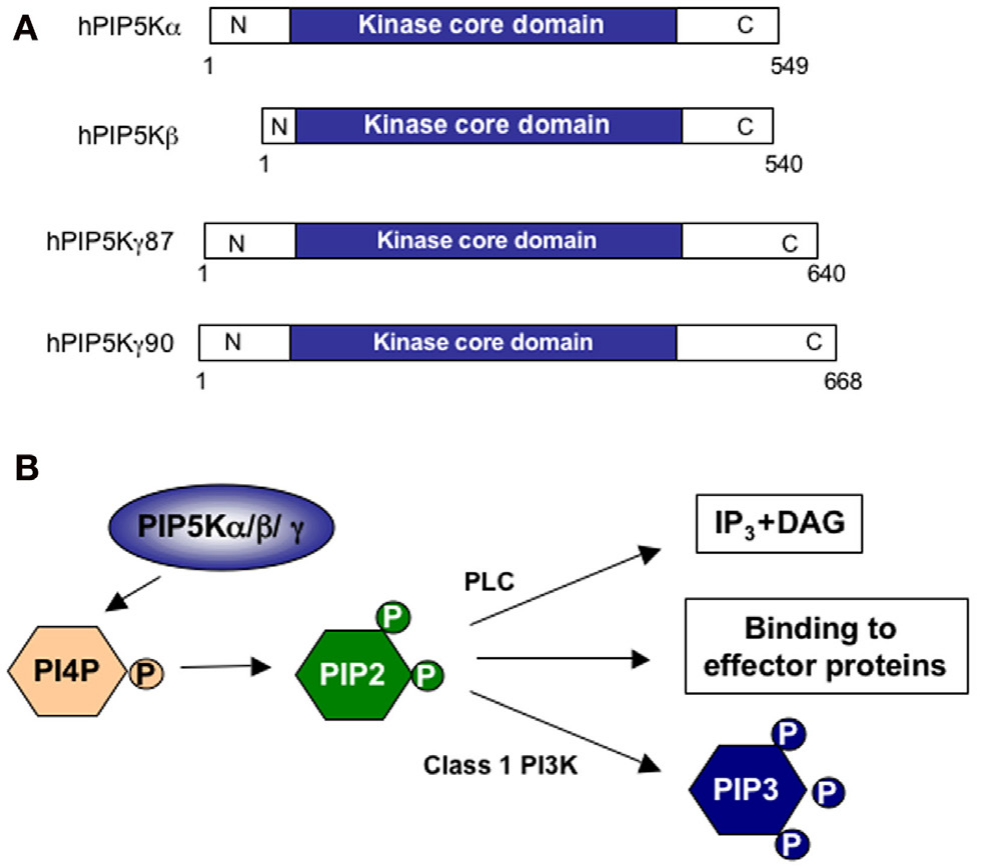
**Structure and functions of PIP5Ks**. **(A)** Schematic representation of human PIP5K (hPIP5K) isoforms with the conserved kinase core domain. **(B)** PIP5K isoforms phosphorylate phosphatidylinositol 4-phosphate (PI4P) in the D5 position of the inositol ring to generate phosphatidylinositol 4,5-bisphosphate (PIP2). PIP2 may be hydrolyzed by phospholipase C (PLC) to generate inositol-1,4,5-trisphosphate (IP3) and diacylglycerol (DAG) second messengers or phosphorylated in the D3 position by class 1 phosphatidylinositol 3-kinase (PI3K) to generate phosphatidylinositol 3,4,5-trisphosphate (PIP3). PIP2 may also directly interact with effector proteins.

Phosphatidylinositol 4,5-biphosphate kinases (PIP5Ks) are a family of isoenzymes that mediate the phosphorylation of phosphatidylinositol 4-phosphate on the D5 position of the inositol ring, thus inducing the production of phosphatidylinositol 4,5-biphosphate (PIP2) (1) (Figure 1). PIP2 is a phospholipid located in the inner leaflet of the plasma membrane that plays a pivotal role in several signaling processes, ranging from the regulation of cytoskeleton dynamics controlling cell migration and cell–cell adhesion to second messenger generation (2, 3). In T lymphocytes, PIP2 regulates the cytoskeleton reorganization events necessary for lymphocyte polarization and migration, the formation of stable T: antigen-presenting cell (APC) conjugates and the further clustering of TCR, co-stimulatory and signaling molecules at the immunological synapse (IS) (4). PIP2 also serves as a precursor for second messengers inositol triphosphate (IP3), diacylglycerol (DAG), and phosphatidylinositol 3,4,5-triphosphate (PIP3) (Figure 1B), which are essential for the activation of the signaling pathways regulating efficient cytokine production, cell cycle progression, survival, and T cell metabolism (5).

Three PIP5K isoforms (α, β, and γ) and further splice variants have been identified (6–8). Primary T cells express all three PIP5K isoforms (9), with differential subcellular localizations, thus providing both temporarily and spatially regulated distinct pools of PIP2 (10–14).

This review illustrates the most relevant functional roles of the different PIP5K isoforms in T cell activation and highlights the molecules and mechanisms involved in their recruitment and activation.

## PIP5K Structure and Activity Regulation

In both humans and mice, all PIP5K isoforms display further variation in their sequence by alternative splicing. Three PIP5Kα, four β, and one γ splice variants have been identified in humans (Figure 1A), and eight PIP5Kα, two β, and three γ splice variants are present in mice. All PIP5K isoforms and splice variants contain a highly conserved kinase domain of 330–380 amino acids with a subdomain, known as the activation loop, which regulates their activity and subcellular localizations (4). The variables N- and C-termini of PIP5K isoforms are also involved in the regulation of lipid kinase activity and in targeting PIP5Ks to specific cellular compartments (3). The last 28 aminoacids of human PIP5Kγ control the interaction of the kinase with talin during the adhesion to the extracellular matrix (15, 16). The C-terminal residues (440–562) of PIP5Kα regulate its localization at nuclear speckles (17). The 83 C-terminal amino acids of PIP5Kβ are essential for its polarization at the uropod (18), whereas the N-terminus controls PIP5Kβ targeting to the plasma membrane and its dimerization with other PIP5K isoforms (19).

Most of the proteins that regulate PIP5K activity belong to the Rho family of small GTPases, which are critical regulators of actin remodeling, vesicular trafficking, and signal transduction (20). In several cell types, Rho, Rac1, and Cdc42 have been reported to interact with all PIP5K isoforms and to activate them in a GTP-independent manner (10, 21).

In addition to Rho, the ADP-ribosylation factor (ARF) GTPases have also been identified as upstream regulators of PIP5K activity at the plasma membrane. ARF GTPases regulate intracellular vesicle trafficking. In particular, ARF6 organizes cortical actin and regulates the traffic between the plasma membrane and the endosomal compartments (22). Several *in vitro* and *in vivo* studies have evidenced a direct role of the membrane-bound ARF6 in activating all PIP5K isoforms (23–25).

All PIP5K isoforms are stimulated by phosphatidic acid (PA), which is generated by phospholipase D (PLD), through the hydrolysis of phosphatidylcholine (26, 27). For instance, both PIP5Kα and PIP5Kγ have been described to interact and colocalize with PLD2 at the membrane to stimulate cell adhesion (28, 29).

The activity of PIP5Ks is also regulated by phosphorylation on Ser/Thr and Tyr residues. For example, phosphorylation of PIP5Kβ at Ser214 (30) and PIP5Kγ at Ser264 (31) in the kinase homology domain have been described as inhibiting PIP5K activity. Phosphorylation of PIP5Kγ on Ser645 inhibits PIP5Kγ interaction with talin (32). Conversely, phosphorylation of tyr644 by Src kinase activates PIP5Kγ (33), whereas tyrosine phosphorylation of PIP5Kβ exerts inhibitory effects (34).

## PIP5Ks and the Regulation of T Cell Polarization, Adhesion, and is Formation

Optimal T cell activation requires the recognition of peptide–MHC by TCR together with co-stimulatory signals, generally provided by counterreceptors expressed on the surface of APCs. CD28 may be considered the most important co-stimulatory molecule. By binding B7.1/CD80 and/or B7.2/CD86, expressed on the surface of activated APCs (i.e., macrophages, dendritic cells, and B lymphocytes), CD28 delivers signals essential for optimal T cell expansion, differentiation, and effector functions (35).

Activation of T cells by APCs bearing the appropriate peptide–MHC complexes initiates with the polarization of membrane receptors and signaling molecules in specific cell locations and is governed by rapid cytoskeletal reorganization events. The dynamic and organization of actin cytoskeleton is tightly regulated by PIP2, which may directly interact with several actin-binding proteins (36), such as talin, vinculin, and filamin (37, 38), thus controlling the selective localization of scaffolding molecules linking the actin cytoskeleton to the plasma membrane (39). Already before interacting with APCs, T cells exhibit a polarized morphology with a leading edge enriched in actin filaments and an uropod enriched in ezrin, moesin, and vimentin filaments (40). The analysis of the distribution of different PIP5K isoforms in mouse T cells revealed that PIP5Kβ and PIP5Kγ90 are predominantly found at the distal pole and in the uropod, thus suggesting a role in adhesion during extravasation from the vasculature (9).

Upon encountering with an APC-bearing specific peptide–MHC complexes, T cells undergo rapid changes in cytoskeletal rearrangements, such as uropod retraction and a strong increase of actin polymerization at the T:APC contact zone. The accumulation of actin and actin-binding proteins in the T:APC contact zone is important for the formation of a stable T:APC conjugate that is necessary for the further clustering of TCR, co-stimulatory, and signaling molecules at the IS (41, 42). Stable conjugate formation requires the interaction between the T cell β2 integrin leukocyte functional antigen-1 (LFA-1) with its ligand intercellular adhesion molecule-1 (ICAM-1) on APCs. LFA-1 activity is regulated by the transition from a low-intermediate to a high-activation state that results in an increase of its affinity for ICAM-1 (43). PIP5Kγ has been described to selectively regulate the affinity of LFA-1 for ICAM-1 by acting downstream of Rho and Rac1 and favoring T cell arrest and stable adhesion (44). More detailed analysis of the role of PIP5Kγ isoforms in knockout mice revealed that CD4^+^ T cells from PIP5Kγ90-deficient mice have increased LFA-1 adhesion to ICAM-1 and T:APC conjugate formation, as well as increased proliferation and cytokine production in response to TCR and CD28 co-engagement (45). Consistent with these data, the two PIP5Kγ isoforms show different cellular localizations during T:APC interaction, with PIP5Kγ87 rapidly, but transiently recruited to the site of T:APC contacts and PIP5Kγ90 in the uropod (9).

Once LFA-1 has mediated a stable contact of T cells with APCs, sustained cytoskeleton rearrangement events occur for the relocalization of receptors, lipid rafts, and signaling complexes at the IS. In particular, the engagement of the TCR and co-stimulatory molecules at the IS promotes the organization of a signaling compartment by inducing cytoskeletal rearrangements and lipid raft accumulation (46–48). All these events are necessary for enhancing TCR-controlled signaling pathways (48–50).

As in other cell types, actin polymerization at the IS is regulated by the Rho family small G proteins, in particular Cdc42 (51), and its guanine nucleotide exchange factor Vav1 (52–55). Following tyrosine phosphorylation and activation, Vav1 promotes the exchange of GDP to GTP and the activation of Cdc42. In the GTP-bound form, Cdc42 interacts with the neuronal Wiskott–Aldrich syndrome protein (N-WASP) that, in turn, binds the actin-related protein (ARP) 2/3 complex to promote actin polymerization (56). The ARP2/3 complex cooperates with filamins in establishing cortical actin architecture (57). Filamin-A is predominantly expressed in the immune system and participates in T cell activation (58, 59). CD28 is the crucial determinant of T lymphocyte activation, as it promotes the cytoskeletal rearrangement events required for the organization of a signaling compartment at the IS (46, 59). CD28 binds and promotes the tyrosine phosphorylation and activation of Vav1 (60–62). CD28 also recruits filamin-A to the membrane, where filamin-A cooperates with Vav1 to integrate signaling pathways resulting in actin polymerization and lipid raft mobilization (59, 63).

**Figure 2:**
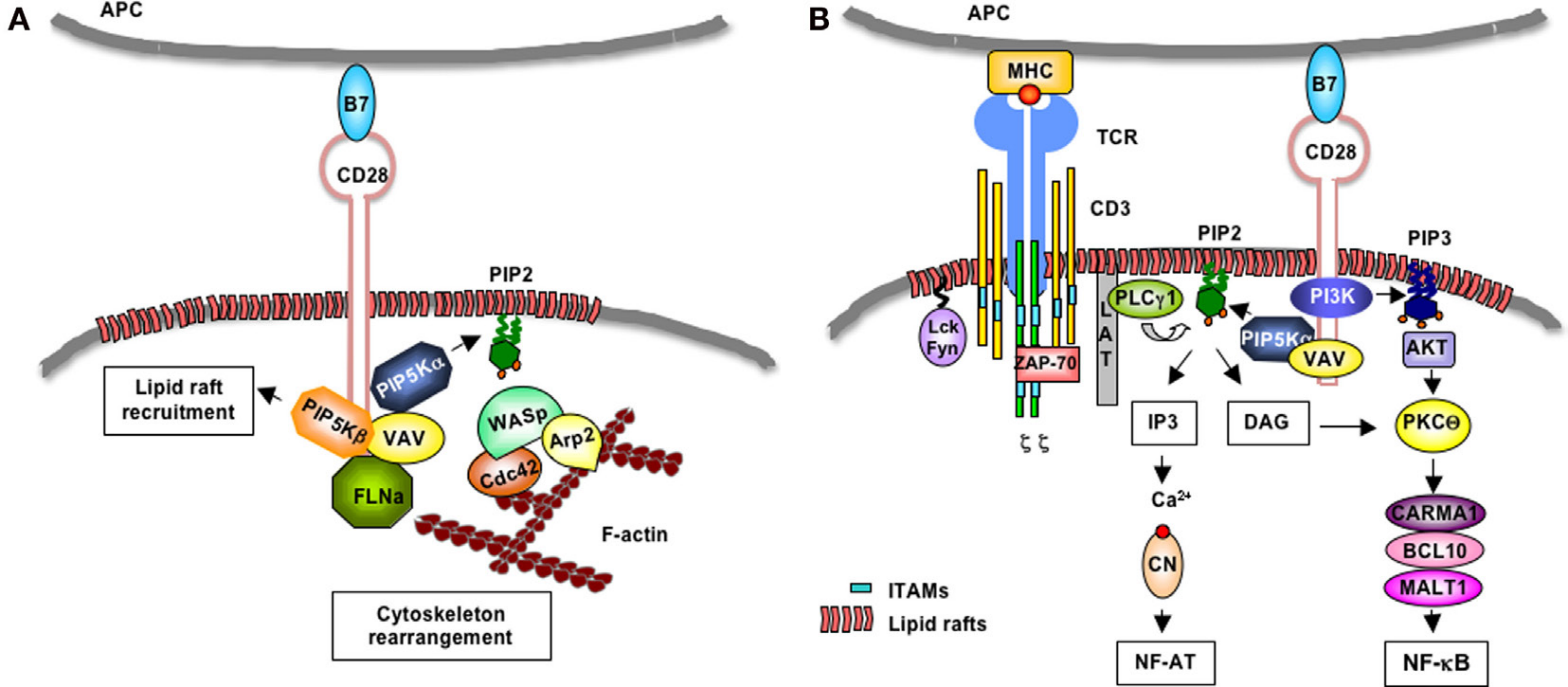
**CD28-induced regulation of PIP5Kα/β at the IS**. **(A)** Following engagement by B7, human CD28 binds Vav1 that in turn favors the recruitment of PIP5Kα and PIP5Kβ. PIP5Kα generates PIP2 that favors the recruitment of the WASP/Cdc42/ARP2/3 complex, which, in turn, promotes actin polymerization. PIP5Kβ mediates the recruitment of filamin-A (FLN-A) and lipid rafts to the T:APC contact zone. **(B)** Upon TCR recognition of peptide–MHC complexes presented on the surface of APCs, Lck and Fyn phosphorylate CD3 and ζ chains, which bind ZAP-70. ZAP-70 phosphorylates LAT that in turn binds PLC-γ1. CD28 mediates the recruitment of Vav-associated PIP5Kα that generates PIP2. PLC-γ1 hydrolyzes PIP2 in IP3 and DAG. IP3 induces the activation of Ca^2+^/calcineurin (CN) and NF-AT. CD28 also binds class 1A PI3K that phosphorylates PIP2 and generates PIP3 that favors the recruitment and activation of Akt. Akt cooperates with DAG to activate PKCθ/CARMA1/Bcl10/MALT1 complex and NF-κB.

Lipid rafts are cholesterol-/sphingolipid-enriched membrane domains, which provide a dynamic lipid environment where critical signaling proteins accumulate (64). Approximately, half of the PIP2 pool associates with membrane rafts (65, 66), which also exhibit locally regulated PIP2 turnover (67). Furthermore, WASP stabilization at the membrane depends on the interaction of its PH domain with PIP2, thus the activity of PIP5Ks is essential for TCR- and CD28-mediated actin reorganization. For instance, recent data from our group evidenced that human PIP5Kβ is recruited to the IS in a CD28-dependent manner and that PIP5Kβ is pivotal for both the recruitment of filamin-A and the accumulation of lipid rafts to the IS. We also demonstrated that PIP5Kβ cooperates with PIP5Kα and Vav1 in promoting actin polymerization and CD28 signaling functions in human T lymphocytes (Figure 2A) (60, 68, 69).

Interestingly, enough, recent data by Choudhuri et al. evidenced a novel function of TCR accumulation at the IS, which involves the TCR sorting and release in extracellular microvesicles that in turn deliver transcellular signals by engaging cognate peptide–MHC on APC (70). Similar mechanisms of exchange of molecules through exosomes and microvesicles during IS have been previously described (71). Since ARF6 and PIP5Ks are crucial regulators of the traffic of vesicles (23–25), it would be interesting to assess the role of PIP5Ks in regulating cell–cell communication through the microvesicles exchange at the IS.

## PIP5Ks and the Regulation of the Calcineurin/NF-AT and NF-κB Signaling Pathways

One key role of PIP2 is to regulate TCR signaling by serving as a substrate for the generation of second messengers. TCR stimulation induces the tyrosine phosphorylation of the immunoreceptor tyrosine-based activation motifs (ITAMs) of CD3 and ζ chains, which in turn bind the Syk family tyrosine kinase Zap-70. Following activation by p56lck and/or p59fyn, Zap-70 phosphorylates the linker for activation of T cells (LAT) that binds and recruits to the membrane the phospholipase C γ1 (PLC-γ1) (72). PLCγ1 hydrolyzes PIP2 into DAG and IP3. While DAG remains in the cellular membrane and activates the RAS/protein kinase C (PKC) θ pathway (73, 74), soluble IP3 induces a strong increase of intracellular Ca^2+^ and the activation of the Ca^2+^/calmodulin-dependent calcineurin. Calcineurin in turn dephosphorylates NF-AT transcription factors (NF-ATc1, c2, and c3 in lymphocytes), thus leading to their translocation into the nucleus, where they bind specific DNA response elements in the promoter of genes critical for T cell functions, such as the IL-2 gene (75). TCR stimulation alone is not sufficient to activate this pivotal signaling pathway and requires the co-engagement of CD28 co-stimulatory molecule (49, 76).

Initial studies by Zaru et al. demonstrated that PIP2 turnover induced by TCR engagement and CD28 co-stimulation was required for sustained Ca^2+^ increase (77). Furthermore, Singleton et al. showed that PIP2 accumulates at the IS during antigen recognition, where it is rapidly consumed by PLCγ1 (78). In response to several receptors, PIP5Kα isoform is recruited to the plasma membrane, where it provides the substrate PIP2 for PLCγ, thus inducing IP3 formation and Ca^2+^ mobilization (79–81). We extended these data to T lymphocytes, by demonstrating that CD28 co-stimulation regulates PIP2 turnover by recruiting and activating PIP5Kα at the IS, thus sustaining TCR-stimulated Ca^2+^ influx and NF-AT nuclear translocation and activation (Figure 2B) (68, 69).

Phosphatidylinositol 4,5-biphosphate 2 also serves as a substrate of PI3K. Although TCR stimulation has been shown to induce PI3K activation (82), CD28 is known to give a major contribution in activating PI3K (83). Indeed, CD28 short cytoplasmic tail contains an N-terminal YMNM motif that, following phosphorylation, binds the p85 subunit of class 1A PI3K (84–86). Class 1A PI3K phosphorylates PIP2 and generates PIP3 (87), which binds the PH domains of several molecules involved in T cell activation, such as phosphoinositide-dependent protein kinase 1 (PDK1), AKT, and Vav1 (88). PDK1 contributes to the canonical NF-κB pathway by associating with caspase recruitment domain membrane-associated guanylate kinase protein 1 (CARMA1) and leading to the membrane recruitment and activation of PKCθ (89, 90). The ternary complex CARMA1/Bcl10/mucosa-associated lymphoid tissue lymphoma translocation protein 1 (MALT1) links TCR to the inhibitor of NF-κB kinase (IKK) α/γ/β complex, thus leading to the phosphorylation-dependent degradation of inhibitor of NF-κB (IκB) and activation of RelA/p50 or c-Rel/p50 dimers (74, 91, 92). CD28 co-segregates with PKCθ to a spatially unique subregion within the IS (93), where it favors the activation of CARMA1/Bcl10/MALT1 complex and IKKs (74). Furthermore, PDK1 recruitment to the membrane also leads to the phosphorylation and activation of Akt (94, 95), which in turn cooperates with PKCθ in stimulating the NF-κB cascade (Figure 2B) (96).

In addition to cooperate with TCR in inducing NF-κB activation, CD28 is also able to autonomously activate IKKα and a non-canonical NF-κB2-like cascade leading to the nuclear translocation and activation of RelA/p52 dimers (55, 97). This CD28 unique signaling to NF-κB converges to the selective regulation of the expression of several genes, including anti- and pro-apoptotic gene of Bcl-2 family (98), the LTR of HIV-1 virus (99), and pro-inflammatory cytokine/chemokines (100, 101). The relevance of PIP5Kα in CD28-dependent NF-κB activation has been recently demonstrated by the impairment of CD28 autonomous signaling regulating NF-κB transcriptional activation and IL-8 gene transcription induced by a lipid-kinase-dead mutant of PIP5Kα in CD4^+^ T cells (60). The intracytoplasmic C-terminal PYAP motif of CD28 is essential for both NF-κB activation and PIP5Kα and β recruitment. Watanabe et al. reported that the substitution of the two proline residues in the C-terminal PYAP motif of murine CD28 strongly reduces NF-κB transcriptional activity (102). Yokosuka et al. further showed that this motif is involved in the recruitment of PKCθ to the IS and its colocalization with CD28 (93). More recently, we evidenced that this motif is also important for the activation of CD28-induced non-canonical NF-κB2-like cascade by binding and recruiting to the membrane IKKα and the IKKα activator NF-κB-inducing kinase (NIK) (63). Our findings that C-terminal PYAP is fundamental for both PIP5Kα and β recruitment strengthens the relevance of PIP5Ks in regulating NF-κB-dependent gene expressions in T lymphocytes.

Finally, PIP5Kα and PIP5Kγ have also been found into the nucleus in specific structures called interchromatin granule clusters or nuclear speckles, where they may regulate pre-mRNA processing and mRNA export (103).

## Potential Role of PIP5Ks in the Regulation of Glucose Metabolism in T Cells

Another important contribution of class 1A PI3K in T cell activation and differentiation is the regulation of glucose metabolism (104). Indeed, upon antigen stimulation, T cells rapidly switch from a catabolic oxidative metabolic state to an anabolic glycolitic metabolic program. By phosphorylating PIP2, class 1A PI3K generates PIP3 lipids that recruit and activate the PDK1/Akt pathway. The PI3K/PDK1/Akt pathway triggers the translocation of the high-affinity glucose transporter 1 (Glut1) from the cytosol to the cell membrane, thus increasing glucose uptake and glycoslysis (105). PI3K/PDK1/Akt also activates the mammalian target of rapamycic complex 1 (mTORC1) that stimulates the activity of several transcription factors, which regulate the expression of genes involved in glycolysis. Moreover, mTORC1-induced upregulation of the glycolytic pathway also favors the differentiation of specific inflammatory Th cell subsets in the periphery (106), in particular, the Th1 and Th17 cell subsets (107), which play a pathogenetic role in several autoimmune diseases (108).

Due to its relevant role in activating class 1A PI3K and sustaining PIP3 levels, CD28 participates in T cell metabolism by enhancing TCR-mediated glucose uptake, aerobic glycolysis, and anabolic pathways (109). Moreover, since PIP2 is an essential limiting factor ensuring the activation of PI3K following CD28 engagement, PIP5Ks may be pivotal in regulating glucose metabolism in T cells. Understanding the role of PIP5Ks in the fine tuning of glucose metabolism in T cells may open new therapeutic approaches for treatment of inflammatory diseases. Interestingly, recent data from PIP5Kα knockout mice revealed faster glucose clearance and resistance to the development of obesity on high fat diet (110).

## Concluding Remarks

The accumulation and consumption of PIP2 in a strictly defined spatiotemporal manner is essential for many cellular processes and, indeed, T lymphocyte triggering is finely tuned by PIP5Ks activity. Emerging studies linking alterations in the metabolism of PIP2 to immune-based diseases suggest that PIP5Ks may represent new therapeutic targets to modulate immunity and inflammation. Interestingly, a crucial role of PIP5Ks in regulating HIV infection has been recently demonstrated. The attachment of HIV-1 to T lymphocytes through CD4/CXCR4 complexes promotes PIP5Kα-dependent PIP2 production, which in turn induces the reorganization of actin cytoskeleton, lipid raft mobilization, and clustering of viral receptors, thus finally leading to membrane fusion and viral core internalization (111). These results, together with the recent identification of one selective inhibitor of PIP5Kα that efficiently inhibits advanced prostate cancer progression (112), indicate that the investigation of PIP5K functions may open up new avenues to novel interesting therapeutic targets for several disorders.

## Author Contributions

All authors listed have made substantial, direct, and intellectual contribution to the work and approved it for publication.

## Conflict of Interest Statement

The authors declare that the research was conducted in the absence of any commercial or financial relationships that could be construed as a potential conflict of interest.

## References

[1] Van Den Bout I Divecha N. PIP5K-driven PtdIns(4,5)P2 synthesis: regulation and cellular functions. J Cell Sci (2009) 122(Pt 21):3837–50. 10.1242/jcs.056127 19889969

[2] Di Paolo G De Camilli P. Phosphoinositides in cell regulation and membrane dynamics. Nature (2006) 443(7112):651–7. 10.1038/nature05185 17035995

[3] Kwiatkowska K. One lipid, multiple functions: how various pools of PI(4,5)P(2) are created in the plasma membrane. Cell Mol Life Sci (2010) 67(23):3927–46. 10.1007/s00018-010-0432-5 20559679 PMC11115911

[4] Tuosto L Capuano C Muscolini M Santoni A Galandrini R. The multifaceted role of PIP2 in leukocyte biology. Cell Mol Life Sci (2015) 72(23):4461–74. 10.1007/s00018-015-2013-0 26265181 PMC11113228

[5] Smith-Garvin JE Koretzky GA Jordan MS. T cell activation. Annu Rev Immunol (2009) 27:591–619. 10.1146/annurev.immunol.021908 19132916 PMC2740335

[6] Ishihara H Shibasaki Y Kizuki N Katagiri H Yazaki Y Asano T Cloning of cDNAs encoding two isoforms of 68-kDa type I phosphatidylinositol-4-phosphate 5-kinase. J Biol Chem (1996) 271(39):23611–4. 10.1074/jbc.271.39.23611 8798574

[7] Ishihara H Shibasaki Y Kizuki N Wada T Yazaki Y Asano T Type I phosphatidylinositol-4-phosphate 5-kinases. Cloning of the third isoform and deletion/substitution analysis of members of this novel lipid kinase family. J Biol Chem (1998) 273(15):8741–8. 10.1074/jbc.273.15.8741 9535851

[8] Loijens JC Anderson RA. Type I phosphatidylinositol-4-phosphate 5-kinases are distinct members of this novel lipid kinase family. J Biol Chem (1996) 271(51):32937–43. 10.1074/jbc.271.51.32937 8955136

[9] Sun Y Dandekar RD Mao YS Yin HL Wulfing C. Phosphatidylinositol (4,5) bisphosphate controls T cell activation by regulating T cell rigidity and organization. PLoS One (2011) 6(11):e27227. 10.1371/journal.pone.0027227 22096541 PMC3214035

[10] Chatah NE Abrams CS. G-protein-coupled receptor activation induces the membrane translocation and activation of phosphatidylinositol-4-phosphate 5-kinase I alpha by a Rac- and Rho-dependent pathway. J Biol Chem (2001) 276(36):34059–65. 10.1074/jbc.M104917200 11431481

[11] Doughman RL Firestone AJ Wojtasiak ML Bunce MW Anderson RA. Membrane ruffling requires coordination between type Ialpha phosphatidylinositol phosphate kinase and Rac signaling. J Biol Chem (2003) 278(25):23036–45. 10.1074/jbc.M211397200 12682053

[12] Barbieri MA Heath CM Peters EM Wells A Davis JN Stahl PD. Phosphatidylinositol-4-phosphate 5-kinase-1beta is essential for epidermal growth factor receptor-mediated endocytosis. J Biol Chem (2001) 276(50):47212–6. 10.1074/jbc.C100490200 11581249

[13] Coppolino MG Krause M Hagendorff P Monner DA Trimble W Grinstein S Evidence for a molecular complex consisting of Fyb/SLAP, SLP-76, Nck, VASP and WASP that links the actin cytoskeleton to Fc{gamma} receptor signalling during phagocytosis. J Cell Sci (2001) 114(23):4307–18. 11739662 10.1242/jcs.114.23.4307

[14] Micucci F Capuano C Marchetti E Piccoli M Frati L Santoni A PI5KI-dependent signals are critical regulators of the cytolytic secretory pathway. Blood (2008) 111(8):4165–72. 10.1182/blood-2007-08-108886 18073347

[15] Di Paolo G Pellegrini L Letinic K Cestra G Zoncu R Voronov S Recruitment and regulation of phosphatidylinositol phosphate kinase type 1 gamma by the FERM domain of talin. Nature (2002) 420(6911):85–9. 10.1038/nature01147 12422219

[16] Ling K Doughman RL Firestone AJ Bunce MW Anderson RA. Type I gamma phosphatidylinositol phosphate kinase targets and regulates focal adhesions. Nature (2002) 420(6911):89–93. 10.1038/nature01082 12422220

[17] Mellman DL Gonzales ML Song C Barlow CA Wang P Kendziorski C A PtdIns4,5P2-regulated nuclear poly(A) polymerase controls expression of select mRNAs. Nature (2008) 451(7181):1013–7. 10.1038/nature06666 18288197

[18] Lacalle RA Peregil RM Albar JP Merino E Martinez AC Merida I Type I phosphatidylinositol 4-phosphate 5-kinase controls neutrophil polarity and directional movement. J Cell Biol (2007) 179(7):1539–53. 10.1083/jcb.200705044 18158329 PMC2373511

[19] Lacalle RA De Karam JC Martinez-Munoz L Artetxe I Peregil RM Sot J Type I phosphatidylinositol 4-phosphate 5-kinase homo- and heterodimerization determines its membrane localization and activity. FASEB J (2015) 29(6):2371–85. 10.1096/fj.14-264606 25713054

[20] Hall A. Rho family GTPases. Biochem Soc Trans (2012) 40(6):1378–82. 10.1042/BST20120103 23176484

[21] Tolias KF Hartwig JH Ishihara H Shibasaki Y Cantley LC Carpenter CL. Type Ialpha phosphatidylinositol-4-phosphate 5-kinase mediates Rac-dependent actin assembly. Curr Biol (2000) 10(3):153–6. 10.1016/S0960-9822(00)00315-8 10679324

[22] Myers KR Casanova JE. Regulation of actin cytoskeleton dynamics by Arf-family GTPases. Trends Cell Biol (2008) 18(4):184–92. 10.1016/j.tcb.2008.02.002 18328709 PMC2885709

[23] Honda A Nogami M Yokozeki T Yamazaki M Nakamura H Watanabe H Phosphatidylinositol 4-phosphate 5-kinase alpha is a downstream effector of the small G protein ARF6 in membrane ruffle formation. Cell (1999) 99(5):521–32. 10.1016/S0092-8674(00)81540-8 10589680

[24] Krauss M Kinuta M Wenk MR De Camilli P Takei K Haucke V. ARF6 stimulates clathrin/AP-2 recruitment to synaptic membranes by activating phosphatidylinositol phosphate kinase type Igamma. J Cell Biol (2003) 162(1):113–24. 10.1083/jcb.200301006 12847086 PMC2172713

[25] Perez-Mansilla B Ha VL Justin N Wilkins AJ Carpenter CL Thomas GM. The differential regulation of phosphatidylinositol 4-phosphate 5-kinases and phospholipase D1 by ADP-ribosylation factors 1 and 6. Biochim Biophys Acta (2006) 1761(12):1429–42. 10.1016/j.bbalip.2006.09.006 17071135

[26] Jenkins GH Fisette PL Anderson RA. Type I phosphatidylinositol 4-phosphate 5-kinase isoforms are specifically stimulated by phosphatidic acid. J Biol Chem (1994) 269(15):11547–54. 8157686

[27] Moritz A De Graan PN Gispen WH Wirtz KW. Phosphatidic acid is a specific activator of phosphatidylinositol-4-phosphate kinase. J Biol Chem (1992) 267(11):7207–10. 1313792

[28] Divecha N Roefs M Halstead JR D’Andrea S Fernandez-Borga M Oomen L Interaction of the type Ialpha PIPkinase with phospholipase D: a role for the local generation of phosphatidylinositol 4, 5-bisphosphate in the regulation of PLD2 activity. EMBO J (2000) 19(20):5440–9. 10.1093/emboj/19.20.5440 11032811 PMC314009

[29] Powner DJ Payne RM Pettitt TR Giudici ML Irvine RF Wakelam MJ. Phospholipase D2 stimulates integrin-mediated adhesion via phosphatidylinositol 4-phosphate 5-kinase Igamma b. J Cell Sci (2005) 118(Pt 13):2975–86. 10.1242/jcs.02432 15976455

[30] Park SJ Itoh T Takenawa T. Phosphatidylinositol 4-phosphate 5-kinase type I is regulated through phosphorylation response by extracellular stimuli. J Biol Chem (2001) 276(7):4781–7. 10.1074/jbc.M010177200 11087761

[31] Aikawa Y Martin TF. ARF6 regulates a plasma membrane pool of phosphatidylinositol(4,5)bisphosphate required for regulated exocytosis. J Cell Biol (2003) 162(4):647–59. 10.1083/jcb.200212142 12925709 PMC2173784

[32] Lee SY Voronov S Letinic K Nairn AC Di Paolo G De Camilli P. Regulation of the interaction between PIPKI gamma and talin by proline-directed protein kinases. J Cell Biol (2005) 168(5):789–99. 10.1083/jcb.200409028 15738269 PMC2171813

[33] Ling K Doughman RL Iyer VV Firestone AJ Bairstow SF Mosher DF Tyrosine phosphorylation of type Igamma phosphatidylinositol phosphate kinase by Src regulates an integrin-talin switch. J Cell Biol (2003) 163(6):1339–49. 10.1083/jcb.200310067 14691141 PMC2173703

[34] Halstead JR Van Rheenen J Snel MH Meeuws S Mohammed S D’Santos CS A role for PtdIns(4,5)P2 and PIP5Kalpha in regulating stress-induced apoptosis. Curr Biol (2006) 16(18):1850–6. 10.1016/j.cub.2006.07.066 16979564

[35] Porciello N Tuosto L. CD28 costimulatory signals in T lymphocyte activation: emerging functions beyond a qualitative and quantitative support to TCR signalling. Cytokine Growth Factor Rev (2016) 28:11–9. 10.1016/j.cytogfr.2016.02.004 26970725

[36] Oude Weernink PA Schmidt M Jakobs KH. Regulation and cellular roles of phosphoinositide 5-kinases. Eur J Pharmacol (2004) 500(1–3):87–99. 10.1016/j.ejphar.2004.07.014 15464023

[37] Fukami K Furuhashi K Inagaki M Endo T Hatano S Takenawa T. Requirement of phosphatidylinositol 4,5-bisphosphate for alpha-actinin function. Nature (1992) 359(6391):150–2. 10.1038/359150a0 1326084

[38] Gilmore AP Burridge K. Regulation of vinculin binding to talin and actin by phosphatidyl-inositol-4-5-bisphosphate. Nature (1996) 381(6582):531–5. 10.1038/381531a0 8632828

[39] Saarikangas J Zhao H Lappalainen P. Regulation of the actin cytoskeleton-plasma membrane interplay by phosphoinositides. Physiol Rev (2010) 90(1):259–89. 10.1152/physrev.00036.2009 20086078

[40] Serrador JM Nieto M Sanchez-Madrid F. Cytoskeletal rearrangement during migration and activation of T lymphocytes. Trends Cell Biol (1999) 9(6):228–33. 10.1016/S0962-8924(99)01553-6 10354569

[41] Huppa JB Gleimer M Sumen C Davis MM. Continuous T cell receptor signaling required for synapse maintenance and full effector potential. Nat Immunol (2003) 4(8):749–55. 10.1038/ni951 12858171

[42] Iezzi G Karjalainen K Lanzavecchia A. The duration of antigenic stimulation determines the fate of naive and effector T cells. Immunity (1998) 8(1):89–95. 10.1016/S1074-7613(00)80461-6 9462514

[43] Kinashi T. Intracellular signalling controlling integrin activation in lymphocytes. Nat Rev Immunol (2005) 5(7):546–59. 10.1038/nri1646 15965491

[44] Bolomini-Vittori M Montresor A Giagulli C Staunton D Rossi B Martinello M Regulation of conformer-specific activation of the integrin LFA-1 by a chemokine-triggered Rho signaling module. Nat Immunol (2009) 10(2):185–94. 10.1038/ni.1691 19136961

[45] Wernimont SA Legate KR Simonson WT Fassler R Huttenlocher A PIPKI gamma 90 negatively regulates LFA-1-mediated adhesion and activation in antigen-induced CD4+ T cells. J Immunol (2011) 185(8):4714–23. 10.4049/jimmunol.1001445 20855869 PMC3014605

[46] Tavano R Gri G Molon B Marinari B Rudd CE Tuosto L CD28 and lipid rafts coordinate recruitment of Lck to the immunological synapse of human T lymphocytes. J Immunol (2004) 173:5392–7. 10.4049/jimmunol.173.9.5392 15494485

[47] Viola A. The amplification of TCR signaling by dynamic membrane microdomains. Trends Immunol (2001) 22(6):322–7. 10.1016/S1471-4906(01)01938-X 11377292

[48] Viola A Lanzavecchia A. T cell activation determined by T cell receptor number and tunable thresholds. Science (1996) 273(5271):104–6. 10.1016/S1471-4906(01)01938-X 8658175

[49] Tuosto L Acuto O. CD28 affects the earliest signaling events generated by TCR engagement. Eur J Immunol (1998) 28(7):2132–42. 10.1002/(SICI)1521-4141(199807)28:07<2131::AID-IMMU2131>3.0.CO;2-Q 9692882

[50] Viola A Schroeder S Sakakibara Y Lanzavecchia A. T lymphocyte costimulation mediated by reorganization of membrane microdomains. Science (1999) 283(5402):680–2. 10.1126/science.283.5402.680 9924026

[51] Cannon JL Burkhardt JK. The regulation of actin remodeling during T-cell-APC conjugate formation. Immunol Rev (2002) 186(1):90–9. 10.1034/j.1600-065X.2002.18609.x 12234365

[52] Acuto O Michel F CD28-mediated co-stimulation: a quantitative support for TCR signalling. Nat Rev Immunol (2003) 3(12):939–51. 10.1038/nri1248 14647476

[53] Michel F Acuto O. CD28 costimulation: a source of Vav-1 for TCR signaling with the help of SLP-76? Sci STKE (2002) 2002(144):e35. 10.1126/stke.2002.144.pe35 12165654

[54] Rudd CE Raab M. Independent CD28 signaling via VAV and SLP-76: a model for in trans costimulation. Immunol Rev (2003) 192(1):32–41. 10.1034/j.1600-065X.2003.00005.x 12670393

[55] Tuosto L NF-kappaB family of transcription factors: biochemical players of CD28 co-stimulation. Immunol Lett (2011) 135(1–2):1–9. 10.1016/j.imlet.2010.09.005 20863851

[56] Muscolini M Camperio C Porciello N Caristi S Capuano C Viola A Phosphatidylinositol 4-phosphate 5-kinase alpha and Vav1 mutual cooperation in CD28-mediated actin remodeling and signaling functions. J Immunol (2015) 194(3):1323–33. 10.4049/jimmunol.1401643 25539813

[57] Schneider H Rudd CE. CD28 and Grb-2, relative to Gads or Grap, preferentially co-operate with Vav1 in the activation of NFAT/AP-1 transcription. Biochem Biophys Res Commun (2008) 369(2):616–21. 10.1016/j.bbrc.2008.02.068 18295596 PMC4186964

[58] Thaker YR Schneider H Rudd CE TCR and CD28 activate the transcription factor NF-kappaB in T-cells via distinct adaptor signaling complexes. Immunol Lett (2015) 163(1):113–9. 10.1016/j.imlet.2014.10.020 25455592 PMC4286576

[59] Lettau M Pieper J Janssen O. Nck adapter proteins: functional versatility in T cells. Cell Commun Signal (2009) 7:1. 10.1186/1478-811X-7-1 19187548 PMC2661883

[60] Stossel TP Condeelis J Cooley L Hartwig JH Noegel A Schleicher M Filamins as integrators of cell mechanics and signalling. Nat Rev Mol Cell Biol (2001) 2(2):138–45. 10.1038/35052082 11252955

[61] Hayashi K Altman A. Filamin A is required for T cell activation mediated by protein kinase C-theta. J Immunol (2006) 177(3):1721–8. 10.4049/jimmunol.177.3.1721 16849481

[62] Tavano R Contento RL Baranda SJ Soligo M Tuosto L Manes S CD28 interaction with filamin-A controls lipid raft accumulation at the T-cell immunological synapse. Nat Cell Biol (2006) 8(11):1270–6. 10.1038/ncb1492 17060905

[63] Muscolini M Sajeva A Caristi S Tuosto L A novel association between filamin A and NF-kappaB inducing kinase couples CD28 to inhibitor of NF-kappaB kinase alpha and NF-kappaB activation. Immunol Lett (2011) 136(2):203–12. 10.1016/j.imlet.2011.01.011 21277899

[64] Simons K Gerl MJ. Revitalizing membrane rafts: new tools and insights. Nat Rev Mol Cell Biol (2010) 11(10):688–99. 10.1038/nrm2977 20861879

[65] Liu Y Casey L Pike LJ. Compartmentalization of phosphatidylinositol 4,5-bisphosphate in low-density membrane domains in the absence of caveolin. Biochem Biophys Res Commun (1998) 245(3):684–90. 10.1006/bbrc.1998.8329 9588175

[66] Pike LJ Miller JM. Cholesterol depletion delocalizes phosphatidylinositol bisphosphate and inhibits hormone-stimulated phosphatidylinositol turnover. J Biol Chem (1998) 273(35):22298–304. 10.1074/jbc.273.35.22298 9712847

[67] Golub T Caroni P. PI(4,5)P2-dependent microdomain assemblies capture microtubules to promote and control leading edge motility. J Cell Biol (2005) 169(1):151–65. 10.1083/jcb.200407058 15809307 PMC2171909

[68] Kallikourdis M Trovato AE Roselli G Muscolini M Porciello N Tuosto L Phosphatidylinositol 4-phosphate 5-kinase β controls recruitment of lipid rafts into the immunological synapse. J Immunol (2016) 196(4):1955–63. 10.4049/jimmunol 26773155

[69] Muscolini M Camperio C Capuano C Caristi S Piccolella E Galandrini R Phosphatidylinositol 4-phosphate 5-kinase alpha activation critically contributes to CD28-dependent signaling responses. J Immunol (2013) 190(10):5279–86. 10.4049/jimmunol.1203157 23589613

[70] Choudhuri K Llodra J Roth EW Tsai J Gordo S Wucherpfennig KW Polarized release of T-cell-receptor-enriched microvesicles at the immunological synapse. Nature (2014) 507(7490):118–23. 10.1038/nature12951 24487619 PMC3949170

[71] Gutierrez-Vazquez C Villarroya-Beltri C Mittelbrunn M Sanchez-Madrid F. Transfer of extracellular vesicles during immune cell-cell interactions. Immunol Rev (2013) 251(1):125–42. 10.1111/imr.12013 23278745 PMC3740495

[72] Zhang W Samelson LE. The role of membrane-associated adaptors in T cell receptor signalling. Semin Immunol (2000) 12(1):35–41. 10.1006/smim.2000.0205 10723796

[73] Li Y Sedwick CE Hu J Altman A. Role for protein kinase Ctheta (PKCtheta) in TCR/CD28-mediated signaling through the canonical but not the non-canonical pathway for NF-kappaB activation. J Biol Chem (2005) 280(2):1217–23. 10.1074/jbc.M409492200 15536066

[74] Wang D Matsumoto R You Y Che T Lin XY Gaffen SL CD3/CD28 costimulation-induced NF-kappaB activation is mediated by recruitment of protein kinase C-theta, Bcl10, and IkappaB kinase beta to the immunological synapse through CARMA1. Mol Cell Biol (2004) 24(1):164–71. 10.1128/MCB.24.1.164-171.2003 14673152 PMC303359

[75] Gwack Y Feske S Srikanth S Hogan PG Rao A. Signalling to transcription: store-operated Ca2+ entry and NFAT activation in lymphocytes. Cell Calcium (2007) 42(2):145–56. 10.1016/j.ceca.2007.03.007 17572487

[76] Michel F Mangino G Attal-Bonnefoy G Tuosto L Alcover A Roumier A CD28 utilizes Vav-1 to enhance TCR-proximal signaling and NF-AT activation. J Immunol (2000) 165(7):3820–9. 10.4049/jimmunol.165.7.3820 11034388

[77] Zaru R Berrie CP Iurisci C Corda D Valitutti S. CD28 co-stimulates TCR/CD3-induced phosphoinositide turnover in human T lymphocytes. Eur J Immunol (2001) 31(8):2438–47. 10.1002/1521-4141(200108)31:8 11500828

[78] Singleton KL Roybal KT Sun Y Fu G Gascoigne NR Van Oers NS Spatiotemporal patterning during T cell activation is highly diverse. Sci Signal (2009) 2(65):ra15. 10.1126/scisignal.2000199 19351954 PMC2694444

[79] Saito K Tolias KF Saci A Koon HB Humphries LA Scharenberg A BTK regulates PtdIns-4,5-P2 synthesis: importance for calcium signaling and PI3K activity. Immunity (2003) 19(5):669–78. 10.1016/S1074-7613(03)00297-8 14614854

[80] Wang Y Chen X Lian L Tang T Stalker TJ Sasaki T Loss of PIP5KIbeta demonstrates that PIP5KI isoform-specific PIP2 synthesis is required for IP3 formation. Proc Natl Acad Sci U S A (2008) 105(37):14064–9. 10.1073/pnas.0804139105 18772378 PMC2544579

[81] Xie Z Chang SM Pennypacker SD Liao EY Bikle DD. Phosphatidylinositol-4-phosphate 5-kinase 1alpha mediates extracellular calcium-induced keratinocyte differentiation. Mol Biol Cell (2009) 20(6):1695–704. 10.1091/mbc.E08-07-0756 19158393 PMC2655244

[82] Ward SG Ley SC Macphee C Cantrell DA. Regulation of D-3 phosphoinositides during T cell activation via the T cell antigen receptor/CD3 complex and CD2 antigens. Eur J Immunol (1992) 22(1):45–9. 10.1002/eji.1830220108 1346114

[83] Ward SG Westwick J Hall ND Sansom DM. Ligation of CD28 receptor by B7 induces formation of D-3 phosphoinositides in T lymphocytes independently of T cell receptor/CD3 activation. Eur J Immunol (1993) 23(10):2572–7. 10.1002/eji.1830231029 8405057

[84] Cai YC Cefai D Schneider H Raab M Nabavi N Rudd CE. Selective CD28pYMNM mutations implicate phosphatidylinositol 3-kinase in CD86-CD28-mediated costimulation. Immunity (1995) 3(4):417–26. 10.1016/1074-7613(95)90171-X 7584133

[85] Prasad KV Cai YC Raab M Duckworth B Cantley L Shoelson SE T-cell antigen CD28 interacts with the lipid kinase phosphatidylinositol 3-kinase by a cytoplasmic Tyr(P)-Met-Xaa-Met motif. Proc Natl Acad Sci U S A (1994) 91(7):2834–8. 10.1073/pnas.91.7.2834 8146197 PMC43465

[86] Truitt KE Hicks CM Imboden JB. Stimulation of CD28 triggers an association between CD28 and phosphatidylinositol 3-kinase in Jurkat T cells. J Exp Med (1994) 179(3):1071–6. 10.1084/jem.179.3.1071 7509360 PMC2191424

[87] Kane LP Weiss A. The PI-3 kinase/Akt pathway and T cell activation: pleiotropic pathways downstream of PIP3. Immunol Rev (2003) 192(1):7–20. 10.1034/j.1600-065X.2003.00008.x 12670391

[88] Lemmon MA. Pleckstrin homology domains: two halves make a hole? Cell (2005) 120(5):574–6. 10.1016/j.cell.2005.02.023 15766521

[89] Lee KY D’Acquisto F Hayden MS Shim JH Ghosh S. PDK1 nucleates T cell receptor-induced signaling complex for NF-kappaB activation. Science (2005) 308(5718):114–8. 10.1126/science.1107107 15802604

[90] Villalba M Bi K Hu J Altman Y Bushway P Reits E Translocation of PKC[theta] in T cells is mediated by a nonconventional, PI3-K- and Vav-dependent pathway, but does not absolutely require phospholipase C. J Cell Biol (2002) 157(2):253–63. 10.1083/jcb.200201097 11956228 PMC2199257

[91] Rebeaud F Hailfinger S Posevitz-Fejfar A Tapernoux M Moser R Rueda D The proteolytic activity of the paracaspase MALT1 is key in T cell activation. Nat Immunol (2008) 9(3):272–81. 10.1038/ni1568 18264101

[92] Tanner MJ Hanel W Gaffen SL Lin X. CARMA1 coiled-coil domain is involved in the oligomerization and subcellular localization of CARMA1 and is required for T cell receptor-induced NF-kappaB activation. J Biol Chem (2007) 282(23):17141–7. 10.1074/jbc.M700169200 17428801

[93] Yokosuka T Kobayashi W Sakata-Sogawa K Takamatsu M Hashimoto-Tane A Dustin ML Spatiotemporal regulation of T cell costimulation by TCR-CD28 microclusters and protein kinase C theta translocation. Immunity (2008) 29(4):589–601. 10.1016/j.immuni.2008.08.011 18848472 PMC2950619

[94] Okkenhaug K Patton DT Bilancio A Garcon F Rowan WC Vanhaesebroeck B. The p110delta isoform of phosphoinositide 3-kinase controls clonal expansion and differentiation of Th cells. J Immunol (2006) 177(8):5122–8. 10.4049/jimmunol.177.8.5122 17015696

[95] Park SG Schulze-Luehrman J Hayden MS Hashimoto N Ogawa W Kasuga M The kinase PDK1 integrates T cell antigen receptor and CD28 coreceptor signaling to induce NF-kappaB and activate T cells. Nat Immunol (2009) 10(2):158–66. 10.1038/ni.1687 19122654 PMC2768497

[96] Bauer B Krumbock N Fresser F Hochholdinger F Spitaler M Simm A Complex formation and cooperation of protein kinase C theta and Akt1/protein kinase B alpha in the NF-kappa B transactivation cascade in Jurkat T cells. J Biol Chem (2001) 276(34):31627–34. 10.1074/jbc.M103098200 11410591

[97] Piccolella E Spadaro F Ramoni C Marinari B Costanzo A Levrero M Vav-1 and the IKK alpha subunit of I kappa B kinase functionally associate to induce NF-kappa B activation in response to CD28 engagement. J Immunol (2003) 170(6):2895–903. 10.4049/jimmunol.170.6.2895 12626540

[98] Cianfrocca R Muscolini M Marzano V Annibaldi A Marinari B Levrero M RelA/NF-kappaB recruitment on the bax gene promoter antagonizes p73-dependent apoptosis in costimulated T cells. Cell Death Differ (2008) 15(2):354–63. 10.1038/sj.cdd.4402264 18034190

[99] Annibaldi A Sajeva A Muscolini M Ciccosanti F Corazzari M Piacentini M CD28 ligation in the absence of TCR promotes RelA/NF-κB recruitment and trans-activation of the HIV-1 LTR. Eur J Immunol (2008) 38(5):1446–51. 10.1002/eji.200737854 18389481

[100] Camperio C Muscolini M Volpe E Di Mitri D Mechelli R Buscarinu MC CD28 ligation in the absence of TCR stimulation up-regulates IL-17A and pro-inflammatory cytokines in relapsing-remitting multiple sclerosis T lymphocytes. Immunol Lett (2014) 158(1–2):134–42. 10.1016/j.imlet.2013.12.020 24412596

[101] Marinari B Costanzo A Marzano V Piccolella E Tuosto L. CD28 delivers a unique signal leading to the selective recruitment of RelA and p52 NF-kappaB subunits on IL-8 and Bcl-xL gene promoters. Proc Natl Acad Sci U S A (2004) 101(16):6098–103. 10.1073/pnas.0308688101 15079071 PMC395929

[102] Watanabe R Harada Y Takeda K Takahashi J Ohnuki K Ogawa S Grb2 and Gads exhibit different interactions with CD28 and play distinct roles in CD28-mediated costimulation. J Immunol (2006) 177(2):1085–91. 10.4049/jimmunol.177.2.1085 16818765

[103] Barlow CA Laishram RS Anderson RA. Nuclear phosphoinositides: a signaling enigma wrapped in a compartmental conundrum. Trends Cell Biol (2010) 20(1):25–35. 10.1016/j.tcb.2009.09.009 19846310 PMC2818233

[104] Finlay DK. Regulation of glucose metabolism in T cells: new insight into the role of Phosphoinositide 3-kinases. Front Immunol (2012) 3:247. 10.3389/fimmu.2012.00247 22891069 PMC3413010

[105] Rathmell JC Fox CJ Plas DR Hammerman PS Cinalli RM Thompson CB. Akt-directed glucose metabolism can prevent Bax conformation change and promote growth factor-independent survival. Mol Cell Biol (2003) 23(20):7315–28. 10.1128/MCB.23.20.7315-7328.2003 14517300 PMC230333

[106] Palmer CS Ostrowski M Balderson B Christian N Crowe SM. Glucose metabolism regulates T cell activation, differentiation, and functions. Front Immunol (2015) 6(22):1. 10.3389/fimmu.2015.00001 25657648 PMC4302982

[107] Delgoffe GM Pollizzi KN Waickman AT Heikamp E Meyers DJ Horton MR The kinase mTOR regulates the differentiation of helper T cells through the selective activation of signaling by mTORC1 and mTORC2. Nat Immunol (2011) 12(4):295–303. 10.1038/ni.2005 21358638 PMC3077821

[108] Galgani M De Rosa V Matarese G. T cell metabolism and susceptibility to autoimmune diseases. Mol Immunol (2015) 68(2 Pt C):558–63. 10.1016/j.molimm.2015.07.035 26265113

[109] Frauwirth KA Riley JL Harris MH Parry RV Rathmell JC Plas DR The CD28 signaling pathway regulates glucose metabolism. Immunity (2002) 16(6):769–77. 10.1016/S1074-7613(02)00323-0 12121659

[110] Huang P Yeku O Zong H Tsang P Su W Yu X Phosphatidylinositol-4-phosphate-5-kinase alpha deficiency alters dynamics of glucose-stimulated insulin release to improve glucohomeostasis and decrease obesity in mice. Diabetes (2011) 60(2):454–63. 10.2337/db10-0614 21270258 PMC3028345

[111] Rocha-Perugini V Gordon-Alonso M Sanchez-Madrid F. PIP2: choreographer of actin-adaptor proteins in the HIV-1 dance. Trends Microbiol (2014) 22(7):379–88. 10.1016/j.tim.2014.03.009 24768560 PMC4171680

[112] Semenas J Hedblom A Miftakhova RR Sarwar M Larsson R Shcherbina L The role of PI3K/AKT-related PIP5K1alpha and the discovery of its selective inhibitor for treatment of advanced prostate cancer. Proc Natl Acad Sci U S A (2014) 111(35):E3689–98. 10.1073/pnas.1405801111 25071204 PMC4156761

